# Expected value and sensitivity to punishment modulate insular cortex activity during risky decision making

**DOI:** 10.1038/s41598-020-68644-5

**Published:** 2020-07-17

**Authors:** Zorina Von Siebenthal, Olivier Boucher, Latifa Lazzouni, Véronique Taylor, Kristina Martinu, Mathieu Roy, Pierre Rainville, Franco Lepore, Dang Khoa Nguyen

**Affiliations:** 1 Département de psychologie, Université de Montréal, Montreal, QC Canada; 2 Centre hospitalier de l’Université de Montréal, Montreal, QC Canada; 3 Centre de Recherche, Institut universitaire de Gériatrie de Montréal, Montreal, QC Canada; 4 Faculté de médicine dentaire, Université de Montréal, Montreal, QC Canada; 5 Alan Edwards Centre for Research on Pain, McGill University, Montreal, QC Canada; 6 Département de Neurosciences, Université de Montréal, Montreal, QC Canada

**Keywords:** Psychology, Neuroscience, Cognitive neuroscience, Emotion, Motivation, Reward

## Abstract

The exact contribution of the insula to risky decision making remains unclear, as are the specific outcome parameters and inter-individual characteristics that modulate insular activity prior to a risky choice. This fMRI study examines the contributions of outcome valence, magnitude, probability, and expected value (EV) to insular activity during risky decision making, and explores the influence of sensitivity to reward and to punishment, and anxiety, to insular activity. Participants (*N* = 31) performed a gambling task requiring choice between two roulettes with different outcome magnitude, probability and EV, under gain and loss conditions separately, and filled questionnaires assessing sensitivity to punishment/reward, and state/trait anxiety. Parametric analyses were conducted to examine the modulation of brain activity during decision making in relation to each task parameter. Correlations were examined between insular activity and psychometric questionnaires. EV of the selected roulette was associated with right posterior insula activation during decision making. Higher sensitivity to punishment was associated with lower bilateral insular activation. These findings suggest that the right posterior insula is involved in tracking the EV of a risky option during decision making. The involvement of the insula when making risky decisions also appears to be influenced by inter-individual differences in sensitivity to punishment.

## Introduction

Decision making is a complex process guided by rational and emotional drives^1,2^. Emotions are particularly determining when facing a risky choice, leading to bias in the decision process^3^. For instance, people tend to weigh losses more heavily than gains and prefer avoiding losses to acquiring objectively commensurate gains, resulting in a greater impact of losses on preferences: a phenomenon known as loss aversion^4^. According to the somatic marker hypothesis, emotions influence the decision making process through "body states”, i.e., internal sensations, visceral, and physiologic changes associated with reinforcing stimuli^5^. The insula is a major cerebral center of visceral sensation processing and interoception (i.e., the sense of the physiological condition of the body)^6^, and is thought to be involved in emotional experience, with some data suggesting a specific response to negative emotions^7–10^. This structure could thus play an important role in risky decision making, but this role remains elusive.

Direct evidence of a contribution of the insula to risky decision-making has been provided by a few lesion studies conducted with small groups of patients with insular damage who showed impaired performance on gambling tasks aimed to simulate everyday decision making situations^11–14^. Using the Cups Task, in which the individual selects between a ‘sure’ option (fixed amount of money) and a risky (varying odds and amount of money) option in order to win (gain condition) or avoid losing (loss condition) money, Weller and colleagues^13^ showed that patients with insular lesions were insensitive to differences in expected value (EV) between choice options. Using the same task with epileptic patients with surgical lesions of the operculo-insular region, our group found reduced sensitivity to EV when facing a potential loss, but not in the gain condition^14^. These results were congruent with the view that partly distinct neural processes are involved during risky decision making depending on whether the potential outcome is a gain or a loss^15–18^, and that the insula is more specifically involved in risky decisions when facing a potential loss^18,19^. However, these lesion studies are limited by heterogeneity of cerebral damage across patients and by the extent of cortical damage to adjoining regions.

Functional neuroimaging studies using gambling tasks have attributed multiple roles to the insula in risky decision making, including risk assessment and prediction error^20^, anticipation of potential gains and losses^21^, outcome processing and feedback integration^22,23^. Using a loss aversion paradigm in which participants had to accept or reject mixed gambles of equal probabilities of gaining or losing different amounts of money, Canessa and colleagues showed that the posterior insula tracks the magnitude of potential losses^24^. While outcome valence, magnitude, probability, and EV may influence activity of the insula during risky decision making, which of these factors specifically influence insular activity remains uncertain. Furthermore, despite evidence that the insula mediates the relationship between emotional state and decision bias^25^, little is known on the inter-individual differences in emotion and risk processing that influence insular involvement in decision making.

The present fMRI study aims to determine the specific contribution of outcome valence, magnitude, probability, and EV to insular activity during risky decision making. Another objective is to examine whether this activity is modulated by interindividiual differences in sensitivity to reward and to punishment, and in trait or state anxiety.

## Methods

### Participants

Thirty-one healthy volunteers (mean age = 27.7 years, SD = 6.6, range 19–51; 15 males) took part in this fMRI study after providing informed and written consent. All of them were right handed, had normal or corrected-to-normal vision, and reported no history of psychiatric or neurological disorders. Participants were recruited using ads published at the University of Montréal. An 80$ financial compensation was given to each participant at the end of the experiment. The study protocol was approved by the ethics committee of the Centre de recherche de l’Institut universitaire de gériatrie de Montréal, and was conducted in accordance with the Declaration of Helsinki.

### fMRI experiment: the roulettes task

**Figure 1:**
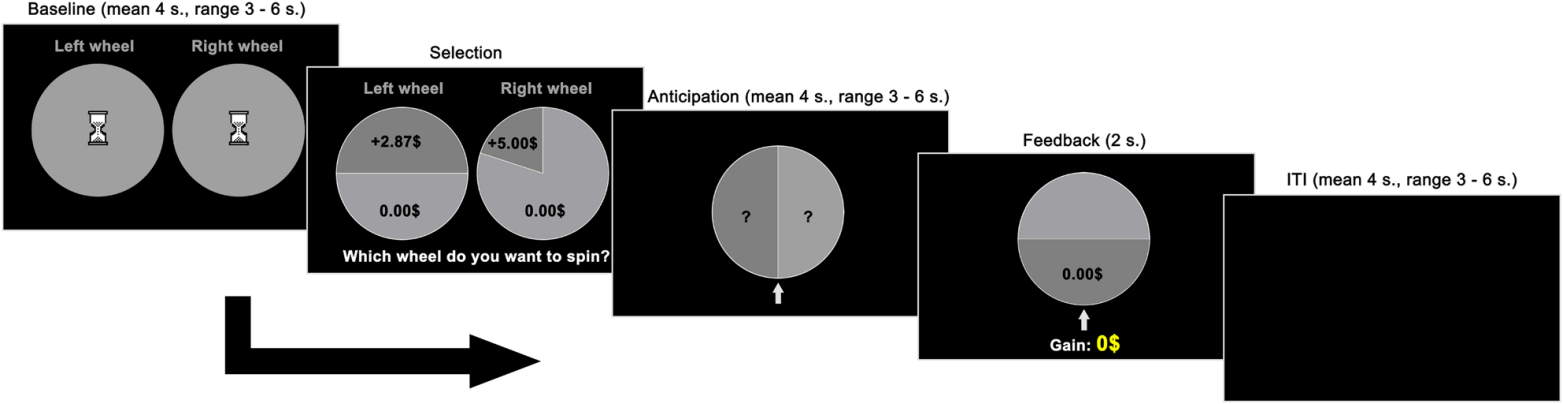
Task design. Each trial is divided in four phases: (1) Baseline, consists of the presentation of two empty wheels on each side of the screen with a question mark at the center; (2) Selection, in which the participant chose one of the two bet options (here, an gain- trial is depicted); (3) Anticipation, in which the selected wheel rotates and (4) Feedback, where the decision outcome was is presented.

Participants performed a computerized roulette task inspired by the Cups Task^13,26^. The task was programmed in E-Prime 2.0 software (Psychology Software Tools, www.pstnet.com). Each trial is divided into four phases: baseline, selection, anticipation, and feedback (see Fig. 1). The Baseline phase consists of the presentation of two empty wheels (roulettes) with a sandglass at the center, presented on each side of the screen for 3–6 s (mean = 4 s). During the Selection phase (i.e., the decision-making phase), the wheels are replaced by new wheels containing two segments. One segment of each wheel shows an amount of money (left wheel: either ± $1.91, ± $2.09, ± $2.87, ± $3.13, ± $4.98, ± $5.02, ± $6.85, ± $7.15, ± $9.79, ± $10.21; right wheel: ± 5.00$), and the other segment has 0$ (we avoided whole numbers in the left wheel to make sure that the two options differed on each trial, and to minimize decisions strictly based on mathematical reasoning). In the left wheel, the segment with an amount of money represents a proportion of 1/10 (10%), 1/7 (14%), 1/5 (20%), 1/3 (33%), or 1/2 (50%) of the wheel, while in the right wheel, it covers 1/5 (20%).

The participant is asked to choose which wheel to spin by pressing the corresponding MRI-compatible button on a keyboard. Half the trials are gain trials, in which the amounts of money are positive; the other half are loss trials, in which the amounts are negative. The Selection phase ends when one bet is selected (i.e., duration of the Selection phase = response time, with no time limit). Then, the wheel is spun at the center of the screen for 3–6 s (mean = 4 s) (Anticipation phase). During the Feedback phase, the wheel stops spinning and the amount of money won/loss is shown on the screen for 2 s. The screen is then left blank for 3–6 s (mean = 4 s) before the next trial. The entire task comprises four blocks of 25 trials each, for a total of 100 trials. In each run, trials are presented in randomised order. Each combination of amount and segment size occurs once for gain and loss trials. At the end of each block, the amount of money gained (or lost) is presented on the screen.

The participant is asked to do the best he/she can to gain as much money as possible in the long run, and is encouraged to respond as he/she would do if using his/her own money. To enhance motivation during the task, the participant is told that he/she will earn the amount of money won during his/her best block at the end of the experiment as a bonus financial compensation, with a maximum of $30, although at the end, each participant is finally given the maximum (i.e., $30) no matter the performance. A few demonstration trials were performed before entering the MRI environment. The percentage of “left” and “right” wheels selected and mean response time were computed separately for the gain and loss conditions. Furthermore, for each trial, an “EV-Based Decision Index” was computed in order to assess at which level the participant based his/her decision according to the EV of each wheel, with the following formula:

$${\text{c}} \cdot \left( {\left| {{\text{EV}}_{{\text{left-adjusted}}} } \right| - \left|{{\text{EV}}_{{{\text{right}}}} } \right|} \right)$$

where “c” is whether the advantageous (according to EV, i.e., absolute value of magnitude x probability) wheel was selected (+ 1 if yes, − 1 if no), “EV_left-adjusted_” is the higher value between EV of the left wheel and 1/EV of the left wheel, and “EV_right_” is the EV of the right wheel (i.e. EV_right_ =  ± 5.00$ * 20% =  ± 1). Thus, a negative or a positive value was given whether the response was disadvantageous or advantageous, respectively, and a larger value was attributed when the difference in EV between the two wheels was larger. According to this formula, more weight is given to trials with larger differences in EV between the two roulettes, no matter which roulette is advantageous. For instance, on trials where the left wheel has 50% chances of winning $10.00, the EV-adjusted of the left wheel is 5 (10 × 50%); the same EV-adjusted is obtained on trials where the left wheel has 10% chances of winning $2.00 (1 ÷ (2 × 10%). By contrast, on a trial with 33.3% chances of winning $3.04, the EV-adjusted of the left wheel is 1.01; the same as on a trial where the left wheel has 20% chances of winning $4.95. Selecting the disadvantageous option in the first cases will lower the EV-Based Decision Index much more than in the second cases. Mean EV-Based Decision Index was computed for gain and loss trials, separately. A positive value insures that the participant considered EVs when making decisions, and did not respond randomly.

### Self-administered questionnaires

Before entering the scanner, participants were asked to complete two psychometric questionnaires. Sensitivity to punishment and to reward was assessed using the French adaptation of the Sensitivity to Punishment and Sensitivity to Reward Questionnaire (SPSRQ)^27,28^. The French adaptation contains 35 items from the original 48-item version; 13 items were removed according to the results of a confirmatory factor analysis^29^, and yes/no ratings were replaced by a 4-point Likert scale, where 1 = “Totally no” and 4 = “Totally yes”, to reduce the bias of Pearson correlation coefficients. Items are divided in two subscales, the Sensitivity to punishment and the Sensitivity to reward scales. The scales are independent and have been shown to have very good internal reliability. The State-Trait Anxiety Inventory (STAI) comprises 40 items, each rated on a 4-point Likert scale where 1 = ‘Not at all’, and 4 = ‘Very much so’^30^. A part of the items are scored reversely to control for positive or negative response biases. A total score (range: 20–80) is computed for state and trait anxiety, separately, and indicates the level of anxiety.

### MRI data acquisition and preprocessing

The functional imaging was conducted by using a 3 T Siemens Trio scanner to acquire gradient echo T2 weighted echo-planar sequence with Blood Oxygenation Level Dependent (BOLD) contrast. The task was projected on a display, which participants viewed through a mirror fitted on top of the head coil. Each image volume corresponds to 51 interspersed axial slices per whole-brain volume at 3 mm thickness, simultaneous excitation of 3 sections (TR = 2,000 ms, TR delay 500 ms, TE = 20 ms, flip angle = 70°, matrix size = 74 × 74, FOV = 220 × 220 mm2, 255/110 volumes; parallel imaging with GRAPPA 2, in-plane resolution = 2.97 X 2.97 mm2, bandwidth = 1732 Hz/Px). The TR delay was included to enable us to record the NFR reflex. The anatomical image was obtained using a high-resolution T1- weighted multi-echo MPRAGE sequence (TR = 2,530 ms; TE = 1.64, 3.50, 5.36, 7.22 ms combined to form one root mean squared (RMS) volume; flip angle = 7°; FOV = 256 mm; matrix = 256 × 256; 1 mm isotropic resolution; 176 slices per whole-brain volume; parallel imaging with GRAPPA 2; bandwidth = 651 Hz/Px).

Preprocessing steps for all subjects included slice time correction of functional data to correct for the differences in image acquisition time between slices, then volumes were aligned to the first to correct for head motion during scanning, after that they were resliced then spatially normalization to the standardized stereotactic space using the Montreal Neurological Institute (MNI) brain template. To finish with spatial pre-processing, images were then convolved in space with a three-dimensional isotropic Gaussian kernel [8 mm full-width half-maximum (FWHM)].

### fMRI data analysis

Functional images were analyzed using the general linear model for block designs in the Statistical Parametric Mapping software (SPM12, Welcome Department of Imaging Neuroscience, London, UK). The statistical analysis of functional data at the within-subject level (first level), where for each subject, changes in brain regional responses were estimated by a general linear model including the responses to the Selection and the baseline conditions (separately and/or pooled for gain and loss trials to the selected wheel), used as regressors to predict brain responses in a block design using the mediation toolbox (https://canlabweb.colorado.edu/wiki/doku.php/help/mediation/m3_mediation_fmri_toolbox)^31^. The model consisted of a boxcar function convolved with the hemodynamic response function (hrf) for each condition. High-pass filtering was implemented in the design matrix using a cutoff period of 128 s to remove slow drifts from the time series. We introduced realignment parameters and mean signal from white matter and cerebro-spinal fluid (using MARSBAR toolbox: https://marsbar.sourceforge.net/) as nuisance regressors in the model. A trial-by-trial parametric analysis was applied to reveal activation modulation of the insula and other areas by decision-choice parameters: EV (magnitude * probability), magnitude, and probability of the selected wheel.

Contrasts of interest were: Selection > Baseline for the selected wheel. This first contrast is used to define the regions relevant to the selection phase. Selection-EV_selected_-Gain, Selection-EV_selected_-Loss, parametric modulation by EV_selected_ for Gain and Loss pooled and separated. Once these contrasts were obtained from first level analysis, they were smoothed and entered in a second level analysis using robust regression^32^ Pearson correlations were also performed with questionnaire scores (i.e., sensitivity to reward, sensitivity to punishment, state anxiety and trait anxiety). Analyses for the contrast Selection > Baseline and for the parametric modulation were performed on the whole brain. We use a bilateral mask on the insula for the examination of the correlation between insular activation and scores to the questionnaires. Insula ROI was defined anatomically using a brain atlas (WKFU_pickatlas software tools was obtained from www.nitrc.org). An anatomically defined ROI was preferred to avoid circularity confound.Contrasts were thresholded using the FDR correction (False Discovery Rate) at q < 0.05. Region labels (> 5 voxels) are reported according to the atlas automated labeling method (aal)^33^, confirmed by visualization of the activation maps over an anatomical image.

### Behavioral data analyses

Behavioral data were analyzed by SPSS 25.0 software (SPSS, Chicago, IL) using descriptive and analytical statistical tests. Pearson correlations were conducted to examine the association between Roulettes Task performance (% of left and right wheels spun in gain and loss conditions separately, and EV-Based Decision Index) and self-administered questionnaires (sensitivity to reward, sensitivity to punishment, trait anxiety, and state anxiety). Gender differences in Roulettes Task performance and self-administered questionnaires scores were examined using univariate analyses of variance (ANOVAs). Correlations and gender differences were considered significant at *p* < 0.05.

## Results

### Behavioral results

Descriptive statistics of the study sample are reported in Table 1. On the Roulettes Task, participants selected the left roulette (with varying amounts, probabilities and EVs) on about half the trials, and the right roulette (with a constant $5.00 amount, 0.20 probability, and EV = 1) in the other half. The value of the EV-Based Decision Index was positive (i.e., > 0.0) for all participants, ensuring that they based their responses on EVs when making decisions, rather than responding randomly. Mean response time did not exceed 4 s. for any participant. Pearson correlations between Roulettes Task performance and self-administered questionnaires can be found in Supplemental Table S1. Increased sensitivity to punishment was associated with a higher number of left roulette selections on loss trials (*r* = 0.33; *p* = 0.045). No other significant correlation emerged. Comparisons according to gender revealed that men tended to report higher sensitivity to reward on the SPSRQ (*F*_(1,29)_ = 4.16, *p* = 0.051), whereas no other difference approached statistical significance (*p*’s > 0.10).

**Table 1:** Descriptive statistics of the study sample (N = 31).

Variable	*N*	Mean ± SD	Range	%
**Sociodemographic characteristics**				
Age (years)	31	27.7 ± 6.6	19–51	
Gender (% male)	15			48.4
**Self-administered questionnaires**				
SPSRQ—sensitivity to reward	31	37.3 ± 7.1	27–55	
SPSRQ—sensitivity to punishment	31	37.9 ± 8.3	21–55	
STAI—state anxiety	30	29.3 ± 8.1	20–52	
STAI—trait anxiety	30	35.3 ± 10.1	21–60	
**Roulette task performance**				
% Left wheel spun—Gain domain	31	51.9 ± 4.6	44–60	
% Left wheel spun—Loss domain	31	45.8 ± 4.4	36–54	
EV-Based Decision Index—Gain domain	31	1.0 ± 0.2	0.1–1.2	
EV-Based Decision Index—Loss domain	31	1.0 ± 0.1	0.7–1.2	
Mean response time—Gain domain (s)	31	1.8 ± 0.5	0.9–3.0	
Mean response time—Loss domain (s)	31	2.0 ± 0.7	0.9–3.7	

### fMRI results

#### Activations during the selection phase minus baseline

**Figure 2:**
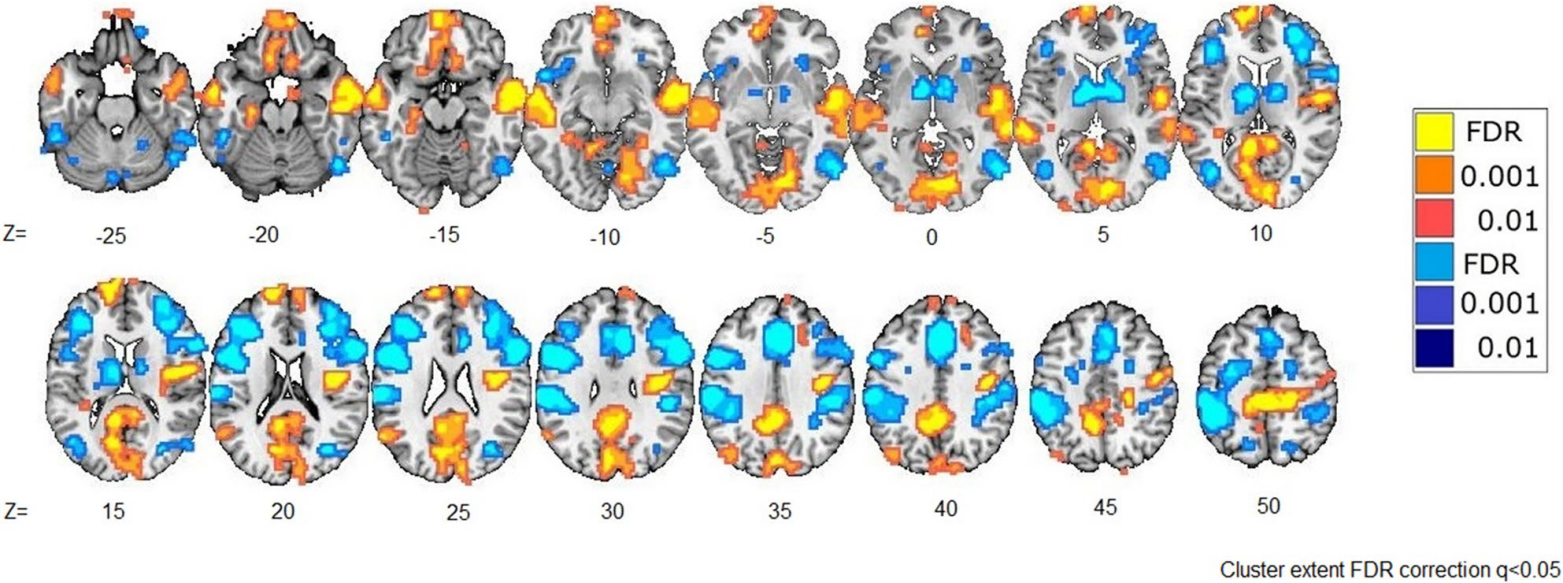
Change in bold activity in the Selection phase in comparison to the Baseline phase. Results are displayed at *q* < 0.05, FDR-corrected. Yellow represents positive effects of selection, and Blue represents negative effects.

Brain activations for the contrast between the roulette Selection phase and Baseline phase are presented in Table 2 and Fig. 2. Maximum insular activity is located in the right posterior insula, although activity is also found in the left insula. Other activations were observed in the frontal (e.g., superior, orbital), temporal (e.g., Heschl, inferior, hippocampus and parahippocampus), parietal (e.g., inferior, precuneus), and occipital (e.g., lingual) regions (Supplemental Table S2).

**Table 2:** Brain activations during decision making: Selection phase minus Baseline phase.

Hemisphere	Anatomical region	MNI coordinates			*Z* score	Cluster size (# of voxels)
		x	y	z		
R	Insula	39	− 10	22	12.28	101
R	Insula	36	− 16	19	5.94	7
L	Insula	− 30	17	4	5.7	6
R	Frontal Sup	12	65	25	7.3	39
R	Frontal Sup	21	32	40	6.1	8
R	Frontal Mid	42	29	22	11.2	910
R	Frontal Mid Orb	21	47	− 20	6.0	7
R	Rectus	9	26	− 17	6.5	19
L	Frontal Sup	− 21	− 7	55	21.5	2,329
L	Frontal Sup Orb	− 18	17	− 14	6.5	6
L	Frontal Med Orb	− 6	62	− 5	10.8	328
L	Frontal Med Orb	− 6	38	− 14	6.6	52
L	Frontal Inf Orb	− 42	17	− 5	7.8	40
L	Frontal Inf Tri	− 42	20	25	13.7	720
R	Heschl	54	− 10	4	13.7	868
R	Temporal Pole Mid	48	20	− 32	5.6	7
R	Temporal Inf	54	− 46	− 26	7.2	58
R	Temporal Inf	51	− 64	− 5	8.8	269
R	ParaHippocampal	33	− 43	− 8	6.5	7
R	ParaHippocampal	21	5	− 29	7.2	10
L	Temporal Mid	− 57	− 13	− 11	9.0	427
L	Temporal Inf	− 51	− 40	− 20	8.7	50
L	Hippocampus	− 24	− 19	− 17	7.5	21
L	Hippocampus	− 30	− 37	− 8	6	7
R	Parietal_Inf	39	− 43	52	12.4	465
R	Precuneus	3	− 55	28	25.6	2,330
L	SupraMarginal	− 54	− 55	25	8	34
L	Angular	− 42	− 73	37	6.6	30
R	Occipital Mid	39	− 70	22	14	71
R	Lingual	12	− 55	4	6.2	27
L	Occipital Mid	− 42	− 70	10	7.5	80
L	Lingual	− 12	− 49	− 8	7	16
R	Cerebellum 6	24	− 55	− 26	10.2	53
L	Cerebellum Crus1	− 39	− 52	− 35	7.4	57
L	Cerebellum 7b	− 33	− 70	− 50	5.6	13
	Vermis 6	0	− 67	− 8	8.4	6
	Vermis 7	3	− 76	− 35	11.4	142

* Cluster extent with FDR correction at *q* value < 0.05.

#### Modulation by task parameters

**Figure 3:**
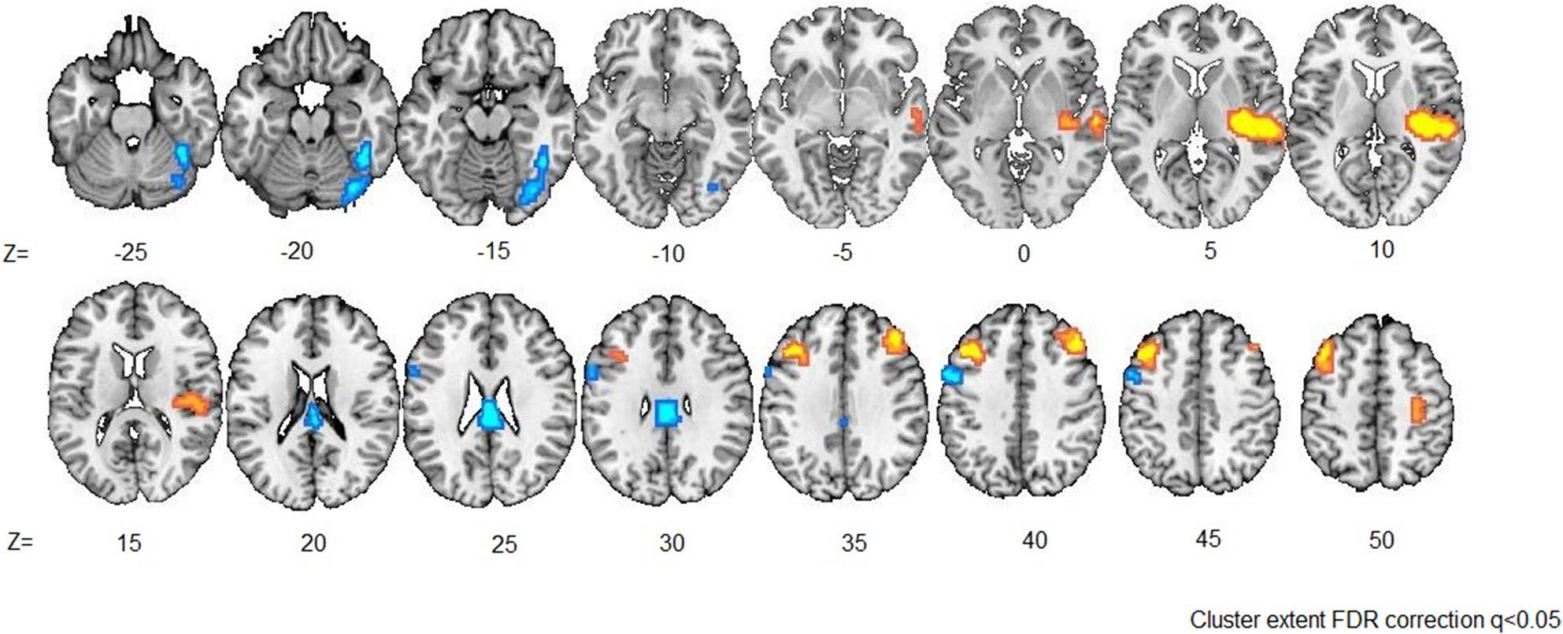
Modulation of BOLD activity by expected value of the selected wheel during the Selection phase. Results are displayed at *q* < 0.05, FDR-corrected. Yellow represents positive effects of modulation, and Blue represents negative effects.

Results from the parametric analysis revealed that activity in the right posterior insula, along with the surrounding Heschl region, right superior temporal gyrus, and left middle frontal gyrus, are positively modulated by the EV of the selected wheel (see Table 3 and Fig. 3). By contrast, insular activity during the roulette selection phase is not significantly modulated by outcome valence, nor by probability or magnitude of the selected wheel. Insular activity modulated by magnitude, probability and outcome valence at an uncorrected threshold of q = 0.01 can be found in Supplemental Table S3.

**Table 3:** Brain activations during the selection phase modulated by EV of the selected wheel.

Hemisphere	Anatomical region	MNI coordinates			*Z* score	Cluster size (# of voxels)
		x	y	z		
R	Insula	36	− 19	7	11.8	20
R	Insula	39	− 19	7	13.7	35
R	Heschl	42	− 22	7	13.7	15
R	Temporal Sup	57	− 25	7	10.3	7
L	Frontal Mid	− 42	26	40	10.4	6

* Cluster extent with FDR correction at *q* value < 0.05.

#### Association between insular activation and inter-individual differences

**Figure 4:**
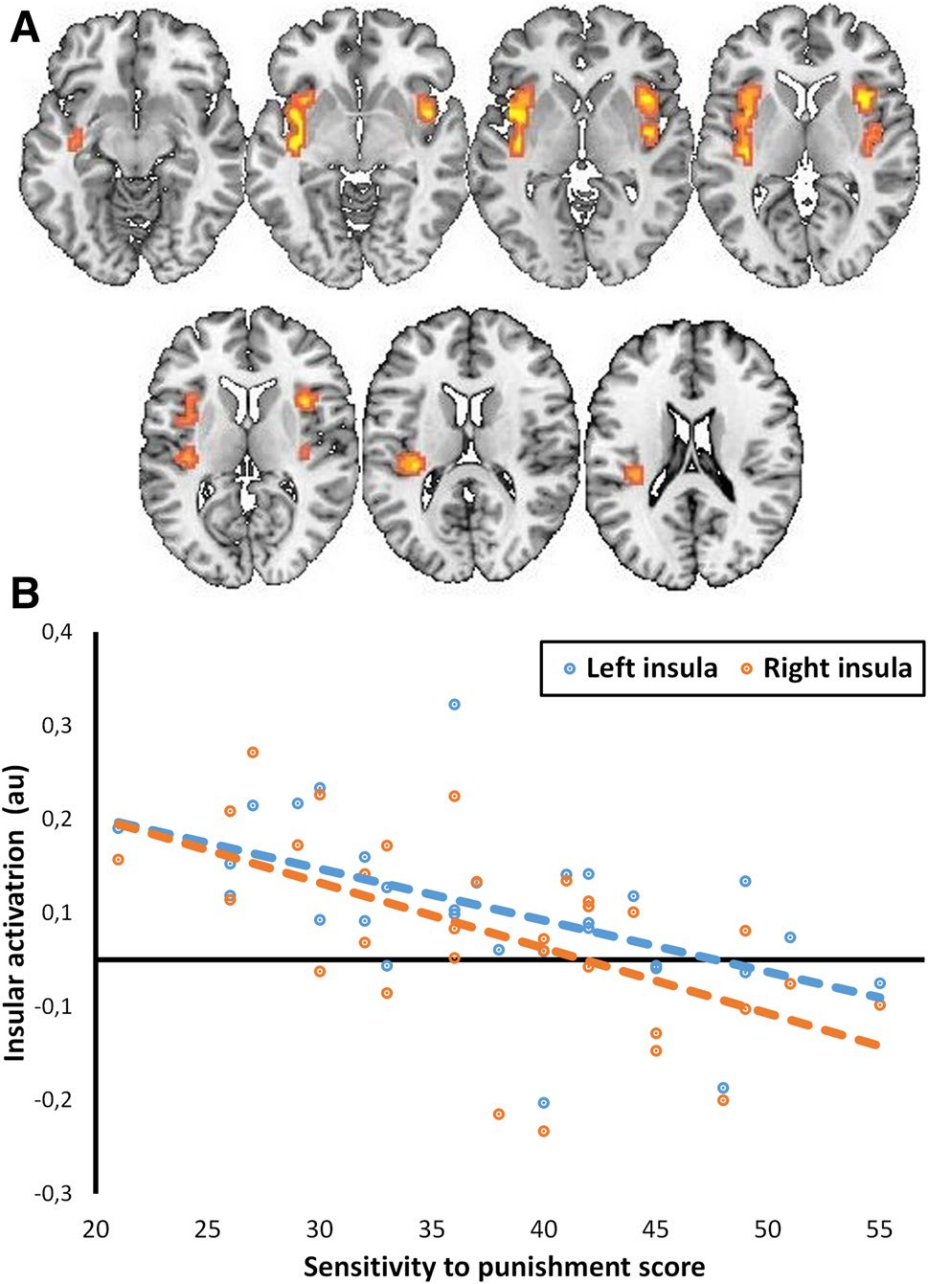
Inter-individual differences in sensitivity to punishment associated with insular activity during the Selection phase. (**a**) Probability maps for bilateral insular activation during the Selection phase, variation as a function of Sensitivity to punishment (results displayed at *q* < 0.05, FDR-corrected). (**b**) Higher scores of Sensitivity to punishment are associated with lower insular activation in the left (*r* = − 0.53, *p* = 0.002) and right (*r* = − 0.58, *p* = 0.001) hemispheres. The colors represent the direction of the effect of selection on the activation of the insula: yellow are positive effects, and blue are negative effects.

Analysis restricted to the insula ROI revealed a significant negative association between bilateral insular activity during the roulette Selection phase and Sensitivity to punishment, independently of outcome valence (Fig. 4). As can be seen in Fig. 4b, higher sensitivity to punishment is associated with lower insular activation during the Selection phase. By contrast, there was no significant association with sensitivity to reward, state anxiety, or trait anxiety.

## Discussion

In this fMRI study, we examined the specific contribution(s) of outcome valence, magnitude, probability, and EV to insular cortex activity during risky decision making, and whether this activity is influenced by inter-individual differences in sensitivity to reward and to punishment, or by trait and state anxiety. Our results suggest that decision making under risk engages the insula (right > left), and independently of outcome valence, and that activity of the right posterior insula is modulated by EV. Furthermore, inter-individual comparisons revealed that higher sensitivity to punishment is associated with decreased activation of the insula bilaterally during risky decision making, regardless of the outcome valence.

Our results add to the existing literature suggesting that the insula is actively involved in risky decision-making^19,20,23,34,35^, and suggest that this involvement is independent of outcome valence. This contrasts with the view that the insula is more especially involved in negative emotion processing^23^, and thus in risky decisions involving a potential loss compared to a potential gain^18,22,24,36^. On the contrary, our findings are concordant with studies that have shown insular activity during both positive and negative rewards (e.g., loss) assessment^22^, during both loss and gain anticipation^21^, and during the selection of a large amplitude reward^37^. Furthermore, lesion studies involving subjects with damage to the insula cortex resulting from stroke have also revealed an impaired decision-making pattern in domains involving both gains and losses^11,13^. Based on results from previous studies, Weller and colleagues proposed that the insula plays a key role by signaling the urge to avoid what is emotionally aversive (e.g., monetary loss), or to obtain a positive emotional state (e.g., monetary gain)^13^.

One of the main findings of the present study is that activity of the right posterior insula during risky option selection is modulated by the EV of the selected option. In a functional neuroimaging study, Rolls and colleagues reported increased insular activity when individuals choose an option with relatively low EV^38^. Lesions studies also suggest a role in sensitivity to EV^11,13,14^, and our results are in striking concordance with those of Weller and colleagues^13^. Using the Cups Task, these authors found that, in contrast to controls, patients with insula damage are unable to adjust their decisions based on EV, i.e., they took as few risks when faced with an advantageous risk proposition (based on the EV) as when faced with a disadvantageous risk propositions. Their study, however, was limited by the fact that patients’ lesions also extended to adjacent regions, which may also have contributed to the deterioration of observed performances. Using the same task, our group found similar findings, although the relative insensitivity to EVs was only observed in the loss domain^14^. Canessa et al. found the posterior insula tracks the magnitude of potential losses during a gambling task^39^. Unlike in our study, probabilities were not manipulated (i.e., each option had 50% probability), so that EV depended solely on magnitude. Our study suggests that the posterior insula tracks EV, rather than solely magnitude, of a selected risky option.

In our study, higher scores on a measure of sensitivity to punishment was associated with decreased activation of the insula during risky decision making, regardless of the outcome valence. This result seems in contradiction with the existing literature suggesting that this region is part of a circuit involved preferably in punishment-based learning^40–43^, and contributes to adapt behavior by choosing a safer subsequent choice, specifically in people prone to anxiety^23^. However, construct validity issues regarding our measure of sensitivity to punishment may account for this surprising finding. Indeed, close examination of the items composing the Sensitivity to punishment subscale of the SPSRQ (e.g., “Do you prefer not to ask for something when you are not sure you will obtain it?”; “Are you easily discouraged in difficult situations?”; “Would it be difficult for you to ask your boss for a raise?”) suggests that scores on this subscale may reflect, at least partly, pessimism rather than sensibility to punishment. Because they expect the worst (i.e., they are less uncertain about the outcome), pessimistic individuals may show reduced anticipatory response while making risky decisions, thereby leading to reduced insular involvement, independently of outcome valence. On the other hand, individuals with a low score on this scale are more optimistic about their gamble, and may be more invested in their decision. This explanation remains speculative and should be explored in future studies. Interestingly, individuals tend to be more optimistic about future gambles after near-miss outcomes, a cognitive distortion that is abolished after insular damage^12^—suggesting that the insula is involved in subjective appraisal of risk and risk prediction error.

In conclusion, this study provides further support for a role of the insula in risky decision making, and contributes to a better understanding of the task and inter-individual factors that modulate insular activity during gamble decisions. Our findings suggest that the right posterior insula is more specifically involved in tracking the EV of a risky option during decision making. Furthermore, the extent of insula cortex involvement in risky decision making appears to be influenced by inter-individual differences in sensitivity to punishment. Combining fMRI with psychophysiological measures in future studies may help better understand the relationship between the somatic state and the neural processes underlying risky decisions.

## Supplementary information

Supplementary file1

## Data Availability

The datasets generated and analyzed during the current study are available from the corresponding author on reasonable request.
