# Supplementary material for: Expected value and sensitivity to punishment modulate insular cortex activity during risky decision making
Source: Sci Rep. 2020 Jul 17;10:11920. doi: 10.1038/s41598-020-68644-5 (PMC7367818; doi:10.1038/s41598-020-68644-5)
Supplement: Supplementary file 1 — Supplementary file1 [file 41598_2020_68644_MOESM1_ESM.docx]

**Expected Value and Sensitivity to Punishment Modulate Insular Cortex Activity During Risky Decision Making**

**Zorina Von Siebenthal, Olivier Boucher, Latifa Lazzouni, Véronique Taylor, Kristina Martinu, Mathieu Roy, Pierre Rainville, Franco Lepore, Dang Khoa Nguyen**

**Supplemental Material**

**Supplemental Table S1.** Pearson correlations between Roulettes Task performance and self-administered questionnaires.

|  | % Left wheel spun | | |  | EV-Based Decision Index | |  | Mean RT | |
| --- | --- | --- | --- | --- | --- | --- | --- | --- | --- |
|  | Gain | Loss |  | | Gain | Loss |  | Gain | Loss |
| SPSRQ – Sensitivity to reward | -0.04 | -0.04 |  | | 0.20 | 0.02 |  | 0.11 | 0.07 |
| SPSRQ – Sensitivity to punishment | -0.00 | 0.33* |  | | 0.13 | 0.04 |  | 0.21 | 0.22 |
| STAI – State anxiety | -0.18 | 0.03 |  | | -0.09 | 0.02 |  | 0.11 | 0.09 |
| STAI – Trait anxiety | -0.21 | 0.06 |  | | 0.07 | 0.04 |  | 0.27 | 0.21 |

***p* < 0.01; * *p* < 0.05.

**Supplemental Table S2.** Whole-Brain activations during the roulette selection phase

| Hemisphere | Anatomical region | MNI coordinates | | | *Z* score | Cluster size (# of voxels) |
| --- | --- | --- | --- | --- | --- | --- |
|  |  | x | y | z |  |  |
| R | Insula | 36 | -22 | 13 | 10.05 | 190 |
| R | Insula | 30 | 23 | -5 | 5.36 | 20 |
| L | Insula | -30 | -28 | 19 | 13.14 | 47 |
| R | Frontal Mid | 42 | 32 | 22 | 11,59 | 551 |
| R | Frontal Mid Orb | 48 | 47 | -14 | 6.05 | 12 |
| R | Frontal Sup | 27 | 50 | 37 | 5,79 | 9 |
| R | Frontal Sup | 27 | 2 | 58 | 7,8 | 213 |
| R | Frontal Sup | 21 | 32 | 40 | 8,07 | 62 |
| R | Frontal Sup Orb | 21 | 44 | -23 | 6,16 | 10 |
| R | Frontal Inf Tri | 57 | 35 | 7 | 6,16 | 9 |
| L | Frontal Mid | -39 | 14 | 52 | 5,08 | 6 |
| L | Frontal Sup | -18 | 2 | 64 | 11,57 | 367 |
| L | Frontal Sup | -18 | 23 | 40 | 8,38 | 33 |
| L | Frontal Sup | -24 | 32 | 46 | 6,22 | 12 |
| L | Frontal Sup Medial | 3 | 26 | 40 | 14,43 | 355 |
| L | Frontal Sup Medial | 0 | 53 | 7 | Inf | 1651 |
| L | Frontal Inf Tri | -42 | 23 | 25 | 16,2 | 496 |
| L | Frontal Inf Tri | -54 | 29 | 4 | 7,25 | 28 |
| L | Temporal Pole Sup | -24 | 5 | -29 | 5,1 | 12 |
| L | Parietal_Inf | -27 | -58 | 37 | 12,64 | 1876 |
| R | Angular | 54 | -61 | 28 | 7,88 | 142 |
| L | Cerebellum Crus1 | -21 | -76 | -35 | 8,82 | 6 |
| R | Cerebellum Crus2 | 33 | -73 | -41 | 6,97 | 167 |
| L | Cerebellum Crus2 | 0 | -82 | -26 | 9,62 | 33 |
| R | Cerebellum 6 | 30 | -55 | -26 | 5,72 | 17 |
| R | Cerebellum 8 | 18 | -73 | -50 | 7,61 | 12 |
| R | Cerebellum 9 | 6 | -49 | -44 | 6,49 | 56 |

* Cluster extent with FDR correction at *q* value *<* 0.05.

**Supplemental Table S3.** Insular activations during the Selection phase modulated by outcome valence, probability, and magnitude of the selected wheel.

| Insula activations modulated by magnitude | | | Uncorrected activations q=0.01 | | |
| --- | --- | --- | --- | --- | --- |
|  | x | y | z | Z score | voxels |
| Right insula | 30 | 20 | -11 | 4.06 | 1 |
| Right insula | 33 | 17 | -8 | 4.22 | 1 |
| Right insula | 33 | 20 | 10 | 4.22 | 2 |
|  |  |  |  |  |  |
| Insula activations modulated by probability | | | | |  |
|  | x | y | z | Z score | voxels |
| Left insula | -30 | 23 | -2 | 4.32 | 1 |
| Left insula | -24 | 20 | 4 | 4.23 | 1 |
|  |  |  |  |  |  |
| Insula activations modulated by valence | | | |  |  |
|  | x | y | z | Z score | voxels |
| Left insula | -33 | -19 | 1 | 4.35 | 8 |
| Left insula | -33 | 14 | 13 | 4.74 | 5 |

* Cluster extent with uncorrected threshold *q* value = 0.01.
